# Hazelnut Allergy

**DOI:** 10.3390/medicina57010067

**Published:** 2021-01-14

**Authors:** Elisabetta Calamelli, Alessia Trozzo, Elisabetta Di Blasi, Laura Serra, Paolo Bottau

**Affiliations:** 1 Pediatric and Neonatology Unit, Imola Hospital, 40026 Imola, Italy; l.serra@ausl.imola.bo.it (L.S.); p.bottau@ausl.imola.bo.it (P.B.); 2 Pediatric Unit, Department of Medical and Surgical Sciences (DIMEC), S.Orsola-Malpighi Hospital, University of Bologna, 40138 Bologna, Italy; trozzoalessia@gmail.com (A.T.); elidiblasi51@gmail.com (E.D.B.)

**Keywords:** hazelnut allergy, component resolved diagnosis, Cor a 9, Cor a 14, lipid transfer protein, oral allergen immunotherapy, pollen food syndrome

## Abstract

*Background and Objectives:* Hazelnuts are frequently involved in IgE-mediated reactions and represent the main culprit of nut allergy in Europe. The clinical presentation varies from mild symptoms limited to the oropharynx [oral allergy syndrome (OAS)], due to the cross-reaction with homologues in pollen allergens and more severe events caused by the primary sensitization to highly stable molecules contained in hazelnuts. The aim of this review is to summarize the most relevant concepts in the field of hazelnut allergy and to provide a practical approach useful in the clinical practice *Materials and Methods:* References were identified by PubMed searches dating from January 2000 up to November 2020 using the search terms: “component resolved diagnosis” and “Hazelnut allergy. *Results:* The storage proteins Cor a 9 and Cor a 14 resulted highly specific for primary hazelnut allergy and strongly associated with severe reactions, while the cross reactive Cor a 1, an homolog of the birch Bet v1, were related to OAS. Any cut-off has shown a specificity and sensitivity pattern as high as to replace the oral food challenge (OFC), which still remains the gold standard in the diagnosis of hazelnut allergy. To date there is still no definitive treatment. Hazelnut free-diet and treatment of symptoms with emergency management, including the prescription of auto-injective epinephrine, still represent the main approach. Oral allergen immunotherapy (AIT) appears a promising therapeutic strategy and the definition of individual clinical threshold would be useful for sensitized individuals, caregivers, and physicians to reduce social limitation, anxiety, and better manage food allergy. *Conclusions:* An accurate diagnostic work-up including clinical history, in vivo and in vitro test including component resolved diagnosis and OFC are essential to confirm the diagnosis, to assess the risk of a severe reaction, and to prescribe an adequate diet and treatment.

## 1. Introduction

Hazelnuts are relevant culprits of food-induced allergy at any age [1]. Hazelnuts belong to the botanical family of Betulaceae, such as trees (birch and adlers), and to genus *Corylus,* species *Corylus avellana*; they are native of Europe and Western Asia, but are also cultivated in North America [1]. The kernel is the edible part of the nut and can be consumed whole, sliced, raw, or roasted. Like other tree nuts, hazelnuts have a great nutrient profile [2,3]. Although high in calories, they are rich in nutrients and their regular consumption has shown to greatly improve the lipid profile, contributing to a decrease of cholesterol levels and, therefore, to a reduction in the risk of coronary heart disease [2,3]. Their potential health benefits are also related to their role in countering the inflammatory processes and oxidative stress, improving glycemic control [2,3]. The renowned benefits of hazelnuts, as well as their pleasant taste, have led to their use in several dishes and in a variety of processed foods (e.g., cookies, cakes, pastries, chocolates, ice creams, breakfast cereals). Nevertheless, hazelnuts also represent a significant source of allergens and their ingestion can induce hypersensitivity reactions in sensitized individuals, varying from mild to potentially life-threatening events [1]. For this reason, hazelnuts with other tree nuts have been included among the potentially allergenic foods which must be labeled to emphasize their content from the rest of the ingredients, regardless of their amount [4,5].

## 2. Aims and Methods

The aim of this review is to summarize the most recent data in the field of hazelnut allergy in order to give some practical indications useful in the clinical practice.

References were identified by PubMed searches dating from January 2000 up to November 2020. Among the 277 references identified using the search terms: “component resolved diagnosis” and “Hazelnut allergy”, 50 were included in this review. For allergen nomenclature, we followed the “Nomenclature system for allergens” that was recommended by the International Union of Immunological Societies (IUIS) [6,7]. This review does not meet the criteria of a systematic review.

## 3. Epidemiology and Clinical Manifestations of Hazelnut Allergy

Hazelnuts represent the main culprit of nut allergy in Europe [8], while walnut and cashew in the USA, and Brazil nut, almond, and walnut are more common in the UK [9,10,11]. The estimated prevalence of hazelnut allergy in Italy and Europe is around 0.2%, [8,12], while the estimated pediatric prevalence of hazelnut allergy in USA and Russia is, respectively, 0.2–0.5% [13] and 0.09% [14]. The high frequency of hazelnut allergy in Italy could be justified by its high consumption rate, considered the highest in the world. A recent Italian epidemiological study conducted by the Italian Society of Pediatric Allergology and Immunology has shown an even greater involvement of tree nuts and, in particular, of hazelnut in anaphylaxis: after milk, tree nuts were the second most frequent cause of generalized allergic reactions, responsible for 16.7% of cases and among all the tree nuts, hazelnut was the leading cause, involved in 40% of cases [15].

Allergy to hazelnut is an IgE-mediated hypersensitivity reaction (type 1) induced by nut proteins, occurring within a few minutes from ingestion [6]. Two clinical pictures of hazelnut-induced IgE-mediated reactions have been described. Primary hazelnut allergy, frequently characterized by generalized systemic and often severe reactions, potentially life threatening, occurring immediately after hazelnut consumption, is due to IgE against specific major hazelnut allergens [1,8]. It is more prevalent in children younger than five years. Severe generalized allergic reactions may be characterized by respiratory (rhinitis and/or asthma), gastrointestinal (abdominal pain, vomiting or diarrhea), and cardiovascular symptoms (tachycardia, hypotension, shock) [6,7]. Indeed, Oral allergy syndrome (OAS), also known as pollen-food syndrome (PFS) is the result of cross-reactivity between homologous proteins contained in both pollens and plant-derived foods [16,17]. PFS manifests with mild symptoms limited to the oropharynx, characterized by itching or burning of the tongue and oral mucosa, and rarely related to anaphylaxis; PFS is typically seen in adolescents and adults with history of seasonal allergic rhinitis [8,16,17,18]. Primary nut allergy and PFS can be usually differentiated by clinical history, even though they could even coexist. Children with primary hazelnut allergy generally present clinical reactions after the first known ingestion of nuts, whereas individuals who develop PFS frequently report no evident adverse manifestations on prior consumption. The site and amount of exposure strongly affect severity of symptoms. For instance, cutaneous exposure is rarely associated to severe adverse reactions, while ingestion of large amounts mainly results in severe response [19]. The frequency and the type of hazelnut-induced allergic reactions seem to vary considerably by geographic region and it is related to the geographical distribution of inhaled cross-reactive pollens (birch/hazel trees). An epidemiological study regarding hazelnut allergy in Europe demonstrated that in the Northwestern and alpine European countries (e.g., Sweden, Germany, The Netherlands, Switzerland) hazelnut allergy is mainly birch pollen related [20,21]. This is due to the homology of protein sequences between Bet v 1 allergen in birch pollen and Cor a 1 allergen in hazelnuts [1,21]. On the other hand, in the Western Mediterranean areas (e.g., Spain, Italy) hazelnut allergy is mainly due to cross-reactivity between the lipid transfer proteins of the peach Pru p 3 and of hazelnut Cor a 8 [1,20]. Furthermore, hazelnut allergy follows age-related specific patterns [21]. Preschool and young scholar children generally present more severe systemic manifestations upon hazelnuts ingestion, mainly due to sensitization to Cor a 9 (hazelnut legumin-like allergen) and unrelated to birch-related allergy. In contrast, adults living in birch endemic areas generally report OAS on hazelnuts intake resulting from the cross-reactivity between hazelnut allergen Cor a 1.04 and birch pollen allergen Bet v 1 [21].

## 4. Diagnosis of Hazelnut Allergy

Diagnosis of hazelnut allergy is based on collection of clinical history, interpretation of sensitization test results (in vitro and in vivo), and execution of open or blinded oral food challenge (OFC) [22].

### 4.1. Clinical History

Before performing any allergy tests, a detailed clinical history should always be the starting point in order to identify suggestive clinical findings compatible with an IgE-mediated reaction, which typically arises immediately (within 2 h) after intake of hazelnut or hazelnut contaminated pre-packaged foods, even if this alone is not sufficient to make a diagnosis of nut allergy. Key points to be investigated include: time interval between exposure and onset of symptoms, clinical manifestations, duration of symptoms, and response to treatment [22,23].

### 4.2. Allergy Tests

The OFC with the culprit food is still considered the gold standard for the diagnosis of food-expensive induced allergy, best if conducted in a double-blind placebo-controlled way [6,8]. Considering its costs, time-consuming and risk of severe reactions, OFC should be performed in an appropriate setting by well-trained personnel: thus, in real life, only few OFC are really performed [6,8,24]. Considering these aspects, the presence of suggestive symptoms of IgE-mediated reaction, and the evidence of hazelnuts specific IgE (sIgE) (positive results on skin prick test or elevated specific IgE), constitute the mainstay of the diagnosis of hazelnut allergy [9]. Skin prick test (SPT), prick-to-prick (PTP) with whole hazelnut, and serum sIgE to hazelnut extract are the key tools to evidence hazelnut IgE sensitization [14]. Some studies indicated that PTP with whole hazelnut seems to be more accurate in predicting hazelnut allergy [25]. Unfortunately the method PTP is not standardized and so not reproducible. Both SPTs and sIgE for whole hazelnut have high sensitivity, but low specificity. Lack of absolute sensitivity is the result of possible allergen alterations in the process necessary to create the extract [26,27], while the limited diagnostic value due to false positive response can be associated to the presence of cross-reactive labile nut allergen protein as Cor a 1 [28,29].

There are few studies assessing the diagnostic values of SPT weal size in tree nut allergy. In the study reported by Ho et al. a SPT ≥ 8 mm for hazelnut had a positive predictive value of 95% for hazelnut allergy. Another study performed by Clark and Ewan involving 1000 children and adults allergic to at least one nut (Brazil nut, walnut, hazelnut, and almond) found that only 3% of the studied population with a SPT ≥ 8 mm was able to tolerate any tree nut and only 5% of them with sIgE value > 15 kU/L tolerated hazelnuts [26]. In another study performed on children, hazelnut sIgE > 15 kU/L had a positive predictive value (PPV) of 57% underling that clinical history is very important to interpreted this result [30]. On the basis of these data, the British Society for Allergy and Clinical Immunology (BSACI) guideline indicates that tree nut allergy is “likely” if the weal of the SPT is ≥ 8 mm in diameter or sIgE is ≥15 kU/L [9], whereas, if the weal diameter of SPT is 3–7 mm, or sIgE < 15 kU/L the test could not be considered conclusive. Therefore, a food challenge may be required to make a definitive diagnosis [9]. 

A component-resolved diagnostic (CRD) test can play an important role to diagnose tree nuts allergy and provides greater accuracy compared to both SPT and sIgE for whole hazelnut. It may be useful to distinguish between primary and secondary sensitization and reduce the number of OFCs [23,31]. CRD avails of purified native or recombinant molecular proteins to detect the sIgE antibody response against individual allergens [32]. CRD has become an important tool for diagnosing food allergy allowing better identification and characterization of the specific molecules involved and improving the specificity of the sIgE testing for the selected food [33]. Nevertheless, CRD also presents some limits. CRD level of sensitivity and specificity is still less accurate compared to the OFC and is expensive if compared to both SPT and extract based sIgE. Additionally, only few relevant allergens have been currently included among the available commercial diagnostic tests (Table 1) [1,34,35]. The majority of molecules considered as food allergens are biochemically classified as proteins or glycoproteins that are naturally present in foods [35]. For hazelnut 10 allergenic molecules, named Cor a 1, Cor a 2, Cor a 8, Cor a 9, Cor a 10, Cor a 11, Cor a 12, Cor a 13, Cor a 14, and Cor a thaumatin-like protein (TLP), have been identified and characterized [1]. The readily-available hazelnut component tests include cross-reactive protein Cor a 1 (PR-10) and Cor a 8 (lipid transfer protein LTP) and the seed storage protein Cor a 9 (11S globulin- legumin), and Cor a 14 (2S albumin) [1,31]. The other components are Cor a 2, a Profilin (similar to Bet v2), Cor a 11 (7 s Globulin-vicillin), Cor a 12 and 13 (oleosins), and Cor a TLP, which is strongly stable to heat and low pH [1,28,36]. Currently available studies indicate the storage proteins Cor a 9 and Cor a 14 as highly specific molecules for primary hazelnut allergy and strongly associated to severe allergic reactions, while the cross reactive protein Cor a 1, an homolog of Bet v1 (PR-10) is instead related to PFS rather than primary severe hazelnut allergy [23]. A recent systematic review and meta-analysis that has evaluated eight studies, seven with data on children and one on a mixed-age population, demonstrated that sIgE to Cor a 9 and Cor a 14 are a good predictor for true hazelnut allergy [36]. This review confirms the cross-reactive nature of Cor a 1 and that the presence of sIgE for this component is bound to birch pollen allergy rather than primary hazelnut allergy. An Italian study on hazelnut-sensitized children including in the systematic review of Nilsson and coworker, showed that sIgE to Cor a 14 was the best tool to discriminate between allergic and tolerant children. Interestingly, in this research the simultaneous presence of sIgE to Cor a 14 and the positivity of PTP with whole hazelnut correctly identified all the children with hazelnut allergy [25]. These data were then confirmed also by Uotila and colleagues in their study [37]. They found that in 82 children and adolescents with suspect hazelnut allergy, sIgE for Cor a 14 and Cor a 9 individually or in association, were the best indicators of true hazelnut allergy [37]. Concluding, the OFC is still the gold standard for the diagnosis of food allergies but is costly, time consuming and risky. Nowadays we have further tools which can provide a more precise indications for performing the OFC. In particular for hazelnut allergy, the combination of suggestive clinical history with well-chosen sIgE test (including sIgE for Cor a 1, Cor a 8, Cor a 9, Cor a 14) can constitute the mainstay of the diagnosis suggesting to perform the OFC only in those patients with discrepancy between clinical history and allergy tests [9,31,36].

## 5. Management and Treatment

Despite the prevalence, severity and impact on the quality of life, to date there is still not a definitive treatment for food-induced allergy. Avoidance of the culprit allergenic food (hazelnut and hazelnut-containing products) and treatment of symptoms with provision of an emergency management plan including the prescription of auto-injective epinephrine still represent the principal means to prevent and treat further adverse reactions in allergic patients [6,9,23]. Nevertheless total avoidance of the offending food is often difficult to achieve: the culprit food may be an essential dietary component and, moreover, it is often hard to identify hidden or cross-reacting allergens. Adrenaline auto-injector prescription is mandatory in the case of previous anaphylaxis; in the case of mild or moderate symptoms the prescription of an adrenaline auto-injector must be individualized and require a precise risk assessment [9,38].

Cross-reactivity is an IgE-mediated immunological response to homologous allergenic molecules and can occur between molecules of closely related species or between highly preserved molecules, with similar biological function, also belonging to different species, called panallergens [28,39,40]. Cross-reactivity between tree nuts and between tree nuts and peanuts was described [9,41]. For hazelnut, the most important allergens sequence similarity are with walnut allergens, such as vicilins (Cor a 11 and Jug r 6: 72%), legumins (Cor a 9 and Jug r 4: 73%), and 2S albumins (Cor a 14 and Jug r 1: 60%), and with the legumin contained in pecan (Cor a 9 and Car 1: 71%) [42].

However, it is important to differentiate cross-sensitivity from cross-reactivity to minimize unnecessary dietary restrictions. The former occurs when the patient has positive SPT and IgE to closely-related food in absence of any clinical manifestation upon food ingestion. While the latter occurs when the patient refers clinical reaction to a closely related food [42]. It could happens because the sequence identity alone may be not sufficient to determine a reaction because the structure of the epitopes may play an important role [42]. In a previous study Maloney et al., showed that even if the 86% of peanut-allergic patients presented a sensitization to tree nuts only 34% have clinical symptoms to tree nuts [43].

Recently, two studies have evaluated the cross-reactivity means of OFC among nuts. The first, the so called NUT CRACKER study, found that the 50% of the patients were allergic to only one or two tree-nuts, Among them the 75% of walnut allergic-patients were allergic to pecan, 83% of cashewnut-allergic patients were also allergic to pistachio, and almost all of pistachio-pecan allergic-patients were also allergic to cashew and walnut, respectively [44]. In addition, the PRONUTS study showed that 60.7% of the involved children had an allergy to more than one seed or nut: 74% of the children with hazelnut allergy were allergic to walnut allergy and 56% of children with walnut allergy had a hazelnut allergy, too. Further data showed that the odds ratio (OR) for coexistent pecan-hazelnut allergy is 14.5, walnut-hazelnut allergy 11.5, hazelnut-sesame seed is 3.6, and peanut-hazelnut allergy is 0.28 [45].

In clinical practice, it frequently happens that physicians advise patients with a single tree nut allergy to follow a total nuts-free diet and to be also careful to avoid pre-packaged foods potentially contaminated by nuts. This option is easier and decreases the risk of reactions due to cross-contamination but at the same time decreases the quality of life of patients. Moreover there is no evidence in excluding tolerated nuts in patients who regularly consume it without history of allergic reactions [9,44,46]. In contrast, the second option, which includes a selective nut-free diet, may increase the quality of life but at the same time may increase the risk of reactions due to contamination or mistake. Finally, another aspect not yet clear is whether the consumption of tolerated nuts in the presence of positive IgE test may increase the risk adverse reactions and cross-reacting or prevent food allergy and potentially confer a tolerance effect for other nuts [44].

As a consequence, both options should be discussed with the patients and their families; the discussion should include a carefully evaluation of potential benefits and risks. The choice of the most suitable option should be individualized and require a precise risk assessment; a complete diagnostic work-up (including the OFC) should be performed to rule out coexisting nuts allergies, aimed to minimize unnecessary dietary restriction, reduce anxiety, and prevent accidental reactions.

In recent years, another therapeutic option is available, so-called Food Allergen Immunotherapy (FA-AIT)-FA-AIT is an active treatment of IgE-mediated food allergy based on regular administration of growing dose of the culprit food in order to achieve desensitization. The primary purpose of this strategy is to increase the threshold of reaction and subsequently to achieve post-discontinuation effectiveness (known as tolerance or sustained unresponsiveness) [47].

Oral route is the most studied form of FA-AIT and the recent EAACI guidelines indicates that Oral AIT “is recommended for persistent cow milk, hen’s egg, or peanut allergy for children from around 4–5 years of age on the basis of its ability to increase the threshold for clinical reactions (grade A)” [47].

The first study on hazelnut oral AIT was published by Morally et al. [48]. The authors demonstrated that, in a population of 100 children (aged 3–9 years), affected by hazelnut allergy, more than one-third well tolerated a challenge with 1635 mg hazelnut protein (eight whole hazelnuts) after six months of oral AIT. Levels of hazelnut sIgE, smaller SPT wheal diameter, older age, and lack of cashew allergy were strongly associated to successful desensitization. The remaining 66% could anyways tolerate twice baseline eliciting dose at six months (median, 259 mg, corresponding to one whole hazelnut), thus gaining protection from accidental exposure even to small hazelnuts quantities hidden in processed food products. Oral AIT was not associated with important adverse reactions [48].

Another strategy to try to improve the quality of life in hazelnut allergy patients is the identification of clinical threshold, i.e., the minimum dose the can elicit a reaction (lowest observed adverse effect level-LOAEL) or the highest dose that does not induce any adverse reaction (no observed adverse effect level -NOAEL). Sensitized individuals can manifest different clinical response to different range of doses after ingestion of foods. As a consequence, execution of a step-wise oral food challenge (OFC) could be an effective approach to establish the individual dose safely tolerated by the patient, avoiding its total elimination. Assessment of clinical thresholds for allergenic foods, especially regarding tree nuts, is still not completely standardized. The study based on DBPCFC reported by Blom et al., was aimed at tracing the individual and population thresholds (LOAEL and NOAEL) after ingestion of five major allergenic foods, including hazelnuts. The DBPCFC was applied to 363 patients (aged 2–16 years), 28 of which presented positive objective symptoms for hazelnut. The authors reported that the threshold dose at which 5% of the hazelnut allergic population presented clinical symptoms was 0.29 mg of hazelnut [49].

A retrospective study aimed to assess the efficacy and tolerability of Hazelnut-Low Dose Oral Food Challenge (H-LDOC) was performed by Barni et al. on 43 hazelnut allergic children/adolescents aged from five to 16 years. The highest quantity of hazelnut safely consumed was 2.5 g. About half of patients had no reactions and the others developed not severe clinical responses (OAS, rash, angioedema, abdominal pain, dyspnea). The study demonstrated that the H-LDOFC (hazelnut- low dose oral food challenge) was safe and associated only with mild adverse reactions, mainly localized symptoms (oral allergic syndrome) [50].

## 6. Conclusions

In summary, hazelnut-triggered IgE-mediated reactions are common at any age, above all in Europe. An accurate diagnostic work-up including clinical history, in vivo and in vitro test including CRD and OFC are essential to confirm the diagnosis, to assess the risk of a severe reaction, and to prescribe an adequate diet. In addition, oral AIT appears a promising therapeutic strategy and the definition of individual clinical threshold would be useful for sensitized individuals, caregivers and physicians to reduce social limitation, anxiety, and better manage food allergy improving quality of life, and preventing potential adverse reactions.

## Figures and Tables

**Table 1:** The molecular allergens available for component resolved diagnosis for hazelnut. Modified from Calamelli et al. [34].

Allergen Source	Biochemical Name		
	Stable Proteins		Labile Proteins
	SSP	LTP	PR-10
Hazelnut *Corylus avellana*	r*Cor a* 9 r*Cor a* 14	r*Cor a* 8	r*Cor a* 1

Legend: SSP: Seed Storage Protein; LTP: Lipid Transfer Protein; PR-10: Pathogenesis-like Protein 10.

## Data Availability

Not applicable.
